# Gurvits Syndrome in the Setting of Diabetic Ketoacidosis: A Case Report and Literature Review

**DOI:** 10.7759/cureus.110563

**Published:** 2026-06-09

**Authors:** Elva Alejandra Manjarrez Granados, Francesca Martinez-Nachon, Mariel Coronel Diaz, Lenny Elizabeth Perez Salgado, Alan Araiza, Joseph Varon

**Affiliations:** 1 Internal Medicine, Dorrington Medical Associates, Houston, USA; 2 School of Medicine, Universidad Autónoma de Baja California, Tijuana, MEX; 3 School of Medicine, Universidad Popular Autonoma del Estado de Puebla, Puebla, MEX; 4 Internal Medicine, Stamford Hospital, Stamford, USA; 5 Internal Medicine/Pulmonology, Independent Medical Alliance, Washington DC, USA

**Keywords:** acute necrotizing esophagitis, black esophagus, candida esophagitis, diabetic ketoacidosis, gurvits syndrome, melena

## Abstract

Acute esophageal necrosis (AEN), also known as “black esophagus,” is a rare but serious clinical condition characterized by diffuse circumferential necrosis of the esophageal mucosa. It is most commonly associated with states of hemodynamic compromise. Diabetic ketoacidosis (DKA) has been identified as a precipitating factor.

We present the case of a 56-year-old female patient with poorly controlled type 2 diabetes mellitus who presented with DKA and melena. Esophagogastroduodenoscopy revealed diffuse circumferential black discoloration of the mid-to-distal esophagus consistent with AEN. Histopathology demonstrated acute inflammation with fungal elements consistent with Candida species. The patient was managed conservatively with intravenous fluids, insulin therapy, proton pump inhibitors, and antifungal treatment, with a favorable clinical outcome.

A review of 11 previously reported cases of DKA-associated AEN demonstrates variable clinical presentation but consistently favorable outcomes. This case supports DKA as a precipitating factor for AEN and suggests a potential role of Candida superinfection as a contributing factor in mucosal injury.

## Introduction

Acute esophageal necrosis (AEN), less commonly referred to as Gurvits syndrome, is an uncommon but clinically significant condition characterized by diffuse circumferential black discoloration of the esophageal mucosa with a sharp demarcation at the gastroesophageal junction [1]. The estimated prevalence is low, reported in less than 0.3% of upper endoscopies, although it is likely underdiagnosed [1,2].

The pathogenesis of AEN is multifactorial and is most often described by a “two-hit” mechanism involving an initial ischemic insult followed by chemical injury from gastric acid exposure [1,3]. This condition has been reported in the context of hemodynamic compromise from cardiovascular diseases, sepsis, or shock [2,3]. Diabetic ketoacidosis (DKA) has been reported as a precipitating factor for AEN in prior case reports [4-14]. In this context, severe dehydration, metabolic acidosis, and hyperglycemia may impair esophageal perfusion and mucosal integrity, contributing to ischemic injury. We present a case of AEN in the setting of DKA complicated by histopathologically confirmed Candida superinfection and provide a literature review to highlight its clinical relevance and potential pathophysiologic implications. 

## Case presentation

A 56-year-old woman with a medical history significant for type 2 diabetes mellitus (T2DM) presented to the emergency department with fatigue, vomiting, and melena. She reported not using her prescribed basal insulin for one week, after which she noticed severe fatigue. Over the prior three days, she had multiple episodes of vomiting, but denied hematemesis or coffee ground emesis. Furthermore, she reported melena over the last two days. She denied any alcohol intake, illicit drug use, or tobacco use.

On presentation, her vital signs were BP 129/99 mmHg, HR 111 beats/min, RR 27 breaths/min, temperature 36.4°C, and SpO₂ 99% on room air. Initial laboratory evaluation demonstrated WBC 21.9 ×10³/µL (reference range: 4.0-11.0 ×10³/µL), hemoglobin 15.7 g/dL (reference range: 12.0-16.0 g/dL), hematocrit 44.3% (reference range: 36-46%), platelets 291 ×10³/µL (reference range: 150-400 ×10³/µL), sodium 123 mmol/L (reference range: 135-145 mmol/L), potassium 3.4 mmol/L (reference range: 3.5-5.0 mmol/L), chloride 90 mmol/L (reference range: 98-107 mmol/L), bicarbonate 11 mmol/L (reference range: 22-29 mmol/L), anion gap 22 (reference range: 8-16), blood urea nitrogen 25 mg/dL (reference range: 7-20 mg/dL), creatinine 1.2 mg/dL (reference range: 0.6-1.1 mg/dL), glucose 611 mg/dL (reference range: 70-99 mg/dL), HbA1c 11.4% (reference range: <5.7%), beta-hydroxybutyrate 6.35 mmol/L (reference range: <0.6 mmol/L), and serum osmolality 289 mOsm/kg (reference range: 275-295 mOsm/kg). Venous blood gas demonstrated a pH of 7.29 (reference range: 7.31-7.41) with a calculated bicarbonate of 12.5 mmol/L (reference range: 22-26 mmol/L). The patient was admitted to the intermediate care unit for DKA consistent with medication nonadherence and initiated on intravenous fluids and insulin infusion per protocol, along with proton pump inhibitor therapy. Although she remained hemodynamically stable throughout hospitalization, significant metabolic derangement consistent with DKA was present.

Within the first 24 hours, her DKA resolved, and she successfully transitioned to subcutaneous insulin. 

**Figure 1:**
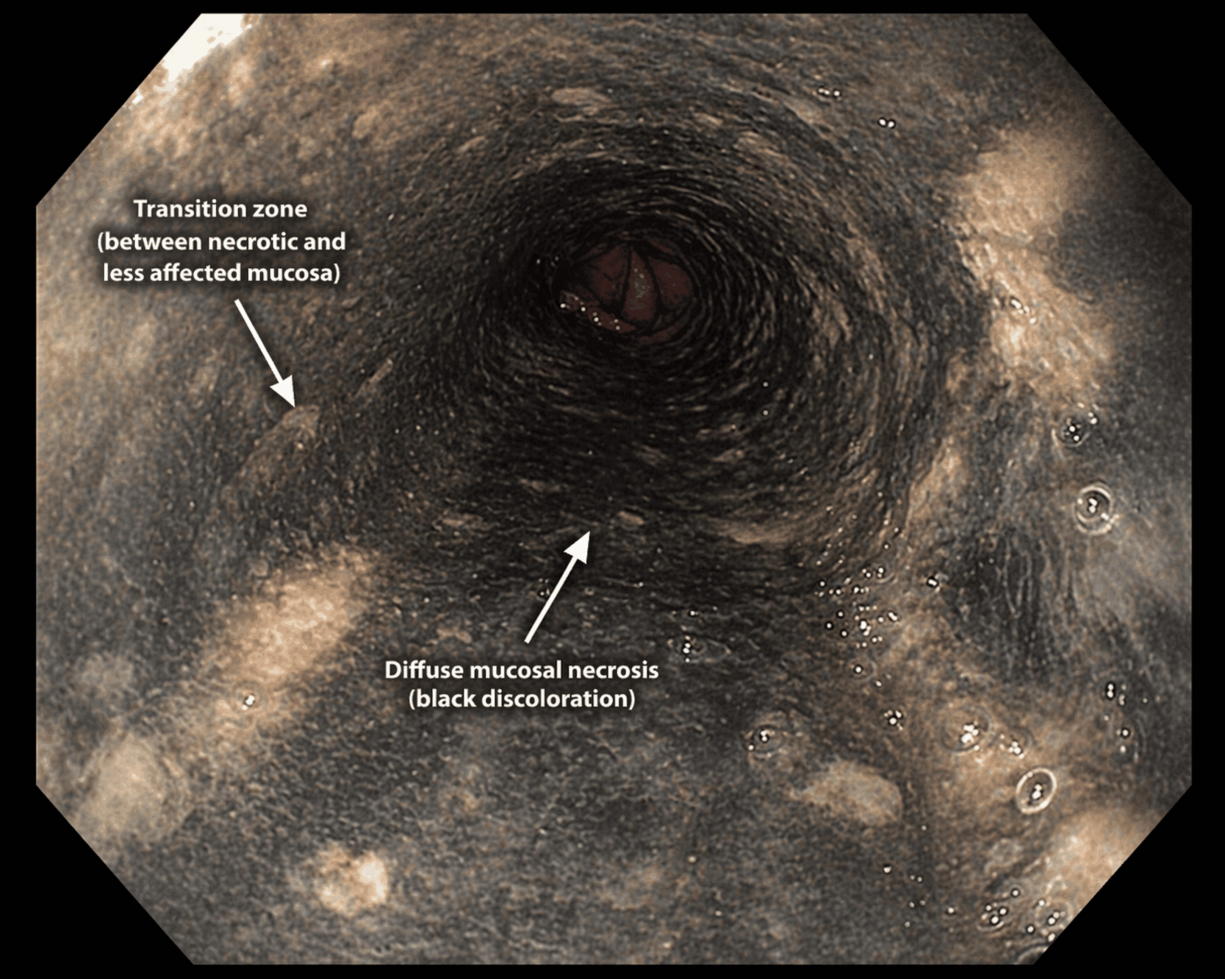
Endoscopic findings consistent with acute esophageal necrosis Esophagogastroduodenoscopy demonstrating diffuse circumferential black discoloration and necrotic-appearing mucosa involving the mid-to-distal esophagus, findings characteristic of acute esophageal necrosis. Arrows highlight areas of diffuse mucosal necrosis and the transition zone between affected and less affected mucosa, with abrupt demarcation of injury.

Due to persistent melena, an esophagogastroduodenoscopy was performed within 48 hours of admission, revealing diffuse circumferential black discoloration involving the mid-to-distal esophagus, consistent with AEN (Figure 1).

The stomach and duodenum were unremarkable. No alternative source of upper gastrointestinal bleeding was identified during endoscopic evaluation. Histopathological analysis demonstrated acute and chronic inflammation with fibrinopurulent exudate. Special stains, GMS and PAS, were positive for Candida species.

The patient was treated with fluconazole for Candida superinfection, along with continued oral proton pump inhibitor therapy and supportive care. Her symptoms improved, the melena resolved, and she was discharged home in stable condition.

## Discussion

This case highlights DKA as a clinically relevant precipitating factor for AEN, a rare but potentially life-threatening cause of upper gastrointestinal bleeding. Despite the severe endoscopic appearance associated with AEN, previously reported DKA-associated cases have generally demonstrated favorable outcomes following prompt metabolic stabilization and supportive management.

We conducted a review of previously reported cases of AEN associated with DKA and identified a total of 11 cases [4-14]. A summary of previously reported cases of DKA-associated AEN is presented in Table 1. These cases included patients ranging in age from 36 to 82 years, with a relatively balanced sex distribution. Clinical presentation varied from hematemesis to melena across reported cases. Upper gastrointestinal bleeding was the predominant presenting manifestation across reported cases, although the severity and specific presentation varied. Interestingly, despite the dramatic endoscopic findings associated with AEN, most patients experienced clinical improvement with conservative management alone, suggesting that DKA-associated AEN may represent a potentially reversible ischemic phenotype when recognized early.

**Table 1:** Reported cases of acute esophageal necrosis associated with diabetic ketoacidosis

Author	Year	Age/Gender	Clinical presentation	Candida	Outcome
Choksi et al. [4]	2017	44-year-old-male	Hematemesis	Not reported	Favorable
Vien et al. [5]	2020	Female (age not specified)	Not reported	Not reported	Favorable
Moss et al. [6]	2021	63-year-old-male	No overt gastrointestinal bleeding	Not reported	Favorable
Valadão et al. [7]	2022	Not specified	Upper gastrointestinal bleeding, unspecified	Not reported	Not reported
Kitawaki et al. [8]	2022	66-year-old-male	Not reported	Not reported	Favorable
Jaber et al. [9]	2023	36-year-old-male	Hematemesis	Not reported	Favorable
Addo et al. [10]	2024	43-year-old-female	Upper gastrointestinal bleeding, unspecified	Not reported	Not reported
Bathobakae et al. [11]	2024	52-year-old-male	Upper gastrointestinal bleeding, unspecified	Yes/Confirmed	Favorable
Tuli et al. [12]	2024	Not specified	Not reported	Not reported	Not reported
Alkhalil et al. [13]	2025	60-year-old-female	Upper gastrointestinal bleeding, unspecified	Not reported	Favorable
Wang et al. [14]	2025	82-year-old-male	Upper gastrointestinal bleeding, unspecified	Not reported	Favorable
Present case	2026	56-year-old-female	Melena	Yes/Confirmed	Favorable
Summary	–	Age range: 36–82 years; median not available	–	Candida reported in 1/11 (9%)	11/11 (100%)

Across these reports, measures of glycemic control such as HbA1c were inconsistently reported, limiting the ability to assess the role of baseline glycemic control in the development of AEN. When available, HbA1c values were markedly elevated (11.4% and 12.2%), supporting poor baseline glycemic control, although these data were only reported in a limited number of cases. This highlights an important gap in the current literature. The limited number of reported cases and inconsistent reporting of clinical variables restrict the ability to draw definitive conclusions regarding prognostic factors and the precise role of contributing mechanisms.

In the setting of DKA, management across reported cases predominantly consisted of conservative therapy, including aggressive fluid resuscitation, insulin administration, and proton pump inhibitor therapy. 

Our case aligns with prior reports, demonstrating a similar pattern of presentation and recovery, with DKA-associated AEN presenting as upper gastrointestinal bleeding and improving with supportive management following metabolic stabilization [4-14]. Notably, this case adds to the limited literature by highlighting the presence of Candida superinfection in DKA-associated AEN, suggesting that Candida superinfection may represent either secondary colonization of necrotic mucosa or a potential contributing factor to ongoing mucosal injury; however, its exact role remains uncertain. The presence of Candida species in AEN remains a subject of clinical debate. While Candida may simply represent colonization of already necrotic mucosa, fungal overgrowth may contribute to ongoing mucosal injury in a compromised epithelial environment [1-3]. In the setting of DKA, where immune function may be impaired, Candida may behave as an opportunistic pathogen in susceptible patients. This raises important clinical considerations regarding the potential role of antifungal therapy, although its routine use remains uncertain.

The pathophysiology of AEN in the setting of DKA is likely driven by a combination of hemodynamic, metabolic, and microvascular factors. Severe dehydration and hypovolemia, commonly seen in DKA, may lead to splanchnic hypoperfusion and ischemic injury to the esophageal mucosa. Additionally, hyperglycemia-associated hyperviscosity and endothelial dysfunction may further impair microvascular blood flow. Ketone bodies and metabolic acidosis may also contribute to direct mucosal injury and disruption of epithelial integrity. Together, these mechanisms likely create a state of critical vulnerability in the esophageal mucosa, predisposing to necrosis even in the absence of overt systemic shock [1,3]. 

Overall, this case further highlights DKA as a clinically relevant precipitating factor for AEN and raises the possibility of a distinct clinical phenotype characterized by reversible metabolic derangement and favorable outcomes when promptly recognized and treated. Further investigation is warranted to better define the role of contributing factors such as microvascular dysfunction and secondary infections, including Candida species [1,3].

## Conclusions

DKA-associated AEN may represent a potentially distinct clinical phenotype characterized by reversible metabolic derangement and favorable outcomes when promptly recognized and treated. Clinicians should maintain a low threshold for endoscopic evaluation in patients with DKA presenting with gastrointestinal bleeding. Further investigation is warranted to better define the role of microvascular dysfunction and secondary infections, including Candida species, in disease progression.

In this case, the patient demonstrated significant clinical improvement following correction of metabolic derangements, supportive care, proton pump inhibitor therapy, and antifungal treatment. She was discharged in stable condition without complications, highlighting the importance of early recognition and multidisciplinary management in DKA-associated AEN.
